# Progress on the BL2 beam measurement of the neutron lifetime

**DOI:** 10.1051/epjconf/201921903002

**Published:** 2019

**Authors:** Shannon F. Hoogerheide, Jimmy Caylor, Evan R. Adamek, Eamon S. Anderson, Ripan Biswas, Sai Meghasena Chavali, Bret Crawford, Christina DeAngelis, Maynard S. Dewey, Nadia Fomin, David M. Gilliam, Kyle B. Grammer, Geoffrey L. Greene, Robert W. Haun, Juliet A. Ivanov, Fangchen Li, Jonathan Mulholland, H. Pieter Mumm, Jeffrey S. Nico, William M. Snow, Daniel Valete, Fred E. Wietfeldt, Andrew T. Yue

**Affiliations:** 1 National Institute of Standards and Technology, Gaithersburg, MD 20899, USA; 2 University of Tennessee, Knoxville, TN 37996, USA; 3 Indiana University, Bloomington, IN 47408, USA; 4 Tulane University, New Orleans, LA 70118, USA; 5 University of Maryland, College Park, MD 20742, USA; 6 Gettysburg College, Gettysburg, PA 17325, USA; 7 Oak Ridge National Laboratory, Oak Ridge, TN 37831, USA; 8 Georgetown University, Washington, DC 20057, USA

## Abstract

A precise value of the neutron lifetime is important in several areas of physics, including determinations of the quark-mixing matrix element │*V*_ud_│, related tests of the Standard Model, and predictions of light element abundances in Big Bang Nucleosynthesis models. We report the progress on a new measurement of the neutron lifetime utilizing the cold neutron beam technique. Several experimental improvements in both neutron and proton counting that have been developed over the last decade are presented. This new effort should yield a final uncertainty on the lifetime of 1 s with an improved understanding of the systematic effects.

## 1. Introduction

Neutron beta decay, the process in which a neutron is transformed into a proton, electron, and electron antineutrino, is well described by the charged weak current model as a left-handed, purely *V*–*A* interaction. Studying the rate at which this process occurs and the angular correlations among the decay products provides insight into this basic semileptonic decay. As the prototypical nuclear beta decay, it is sensitive to certain Standard Model extensions in the charged-current sector. While the most precise determination of the Cabibbo-Kobayashi-Maskawa (CKM) matrix element │*V*_ud_│ is currently obtained from 0^+^ → 0^+^ nuclear decays, it can also be determined via neutron decay through increasingly precise measurements of the neutron lifetime and either the beta asymmetry coefficient [1, 2] or the electron-antineutrino angular correlation coefficient [3, 4]. The reliability of the experimental values is essential for accurate tests of the Standard Model. The neutron lifetime is also an important component in the prediction of the ^4^He mass fraction in the early universe from Big Bang Nucleosynthesis models.

**Figure 1.**
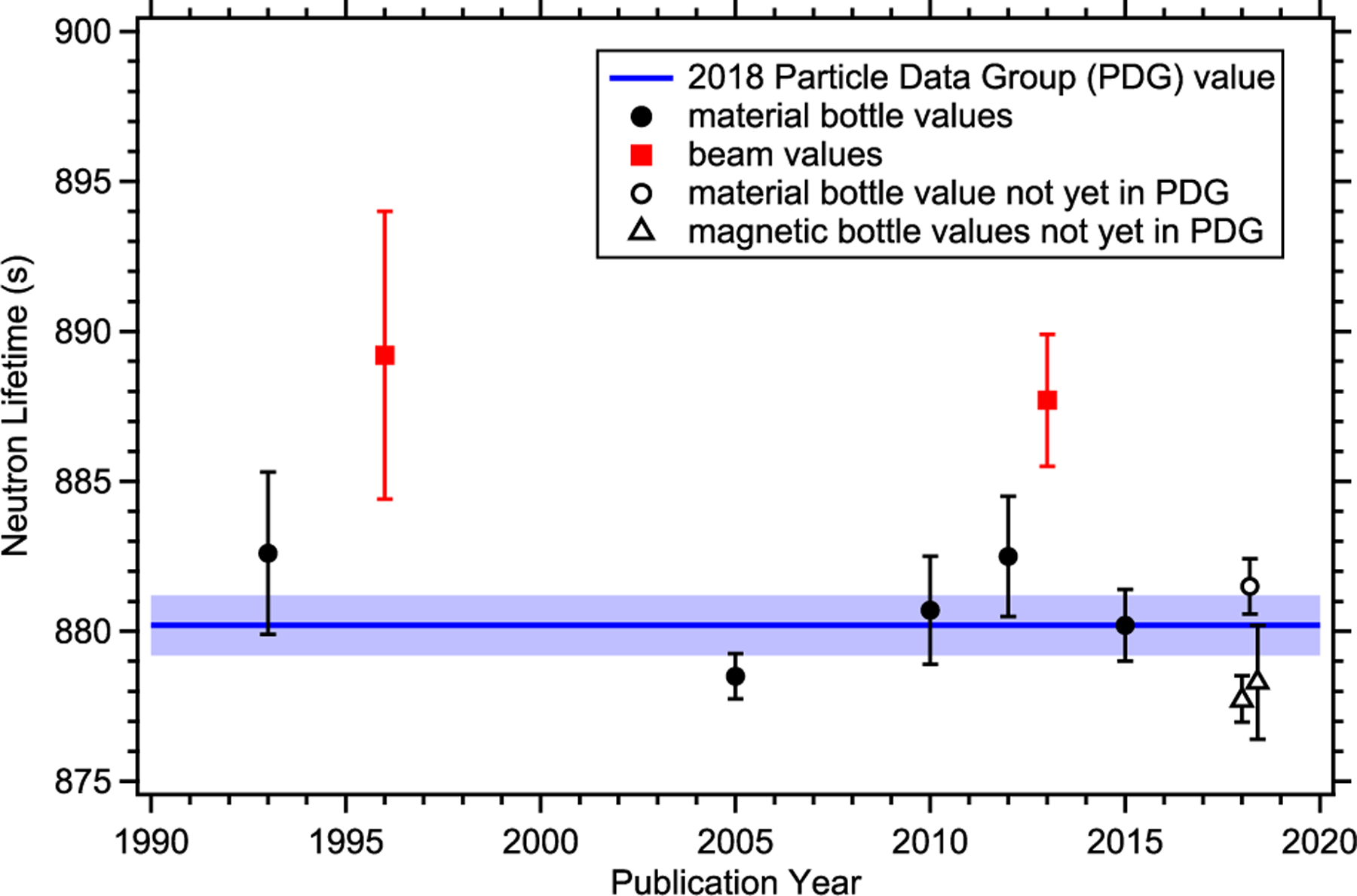
Plot of recent lifetime measurements; all uncertainties are one standard errors. Beam values are shown in solid red squares [11, 15] while UCN material bottle values are shown in solid black circles [16–20]. The 2018 Particle Data Group (PDG) evaluation is shown by the blue line and shaded band. The open circles (material confinement) [21] and open triangles (magnetic confinement) [22, 23] are bottle values not yet included in the PDG evaluation.

The current state of the neutron lifetime is shown in Fig. 1. The value from the 2018 Particle Data Group (PDG) evaluation, shown as the blue line and shaded band in the figure, is 880.2s ± 1.0 s, which includes a scale factor of 1.9 due to the spread in values between the cold neutron beam measurements and the ultracold neutron (UCN) bottle measurements [5]. The PDG value currently includes only the seven values published before 2016 (filled points in Fig. 1). The discrepancy between these reported beam and bottle measurements has prompted significant work in this field, both in experimental efforts as well as attempts at theoretical calculations [6] and exotic explanations such as dark matter [7] or mirror neutrons [8]. In particular, three UCN bottle measurements, utilizing both material and magnetic confinement, have been published since 2016 (open circles in Fig. 1), with more work underway. However, there has been no new measurement of the neutron lifetime using a cold neutron beam since the result from an experiment performed at the National Institute of Standards and Technology (NIST) around 2000. The result of that experiment was re-evaluated based on an improvement in the absolute neutron counting (utilizing the Alpha-Gamma method [9, 10] as discussed in Sect. 3), yielding a lifetime of *τ_n_* = (887.7 ± 1.2[stat] ± 1.9[syst]) s [11]. Currently, there are two experimental efforts underway utilizing cold neutron beams: an upgraded version of the NIST measurement, which is the subject of this paper, and a new campaign underway at the Japan Proton Accelerator Research Complex (J-PARC) that is using a time-projection chamber in order to measure the neutron fluence and rate of decay products [12–14].

In this paper we give an overview of the effort currently underway at NIST to measure the neutron lifetime using a cold neutron beam. This effort is motivated both by the need to address the difference between the lifetime methods and by the progress made in absolute neutron counting, which makes an overall uncertainty of 1 s a realistic goal. We briefly discuss neutron counting improvements including the status of the Alpha-Gamma device, followed by improvements in proton counting and other aspects of the experiment. We conclude with the status and outlook of this experiment.

## 2. The beam method

An in-beam measurement of the neutron lifetime requires the simultaneous counting of both the neutrons in the beam and their decay products. This requires accurate knowledge of both neutron and proton (and/or electron) detector efficiencies as well as the neutron decay volume. The cold neutron beam experiment underway at NIST, BL2, is based on a technique pioneered by Byrne and Greene [15, 24, 25] and utilizes much of the same apparatus as the previous cold neutron beam experiment carried out at NIST (which we shall call BL1) [11, 26, 27]. A segmented electrode stack placed inside a superconducting solenoid is used to trap decay protons. The first three and last three segments of the trap can be held at voltage (typically +800 V) while a variable number of middle segments are grounded. When a neutron decays inside the trap volume, the proton is trapped axially by the applied electrostatic potential, and radially by a 4.6 T magnetic field. After a variable length of time, the front three electrodes are rapidly grounded and a small ramp voltage is applied to the middle electrodes in order to eject the decay protons from the trap and direct them to a silicon proton detector. This detector is held at high voltage (–30 kV) in order to accelerate the low energy protons through the dead layer and allow for their detection. The absolute number of neutrons passing through the decay volume is determined using a neutron flux monitor, located downstream of the proton trap, as described in the next section. The main technical difficulties of our type of beam experiment include the low rate of decay protons, accurate knowledge of the neutron fluence through the decay volume, and a precise measurement of the decay volume itself. As the slow neutron absorption cross section and the amount of time the neutron spends inside the decay volume are both inversely proportional to the neutron’s velocity, a precise measurement of the neutron’s wavelength spectrum is not necessary.

## 3. Neutron counting

The neutron flux monitor consists of a thin, well- characterized ^6^LiF deposit surrounded by 4 silicon charged particle detectors in a precisely defined geometry. Neutron counting is accomplished by counting the alpha and triton particles produced as the neutron beam passes through the ^6^Li. The efficiency of this detector, *ϵ*_0_, is critical to an accurate determination of the neutron lifetime. The uncertainty in BL1 was dominated by uncertainties in systematic effects related to *ϵ*_0_ [26, 27]. The efficiency was derived from the equation

(1) $$\epsilon_{0}=\frac{2N_{A}\sigma_{0}}{4\pi A}\iint \Omega (x,y)\rho (x,y)\phi (x,y)dxdy,$$

which depends on the measured detector solid angle, Ω, the measured neutron beam profile, 𝜙, the measured areal density of the deposit, *ρ*, and the evaluated thermal neutron cross section of ^6^Li, *σ*_0_. *N_A_* is Avogadro’s number and *A* is the atomic weight of ^6^Li. The value of the cross section and uncertainty for the reaction ^6^Li(*n*,*t*) must be taken from evaluated nuclear data files, the latest of which is ENDF/B-VII.0 [28]. This left our result dependent on an evaluated cross section; whenever it was updated, our result needed to be updated. What was worse, the evaluation of this particular cross section is based upon measurements that span a wide neutron energy range and that include no direct measurements made at thermal energies. In order to eliminate this reliance on tabulated data from other experiments, we developed the Alpha-Gamma method [9, 10, 29], a brief overview of which is given here.

The Alpha-Gamma device is a totally absorbing neutron detector based on neutron absorption by ^10^B. It measures neutron fluence by counting gamma-rays from the reaction *n* + ^10^B → ^4^He + ^7^Li^∗^ + *γ* (478 keV) with two calibrated HPGe gamma detectors [10]. The gamma detectors are calibrated in a multi-step procedure that uses a precisely calibrated Pu alpha source, an integrated alpha particle detector, a monochromatic neutron beam, and a thin ^10^B deposit. In regular operation, the thin deposit is replaced with a thick one and the detector operates as a black detector counting the number of neutrons impinging on the deposit per second. “Thin” refers to a deposit from which the alphas can escape while “thick” refers to a deposit capable of stopping all neutrons.

The lifetime experiment’s neutron flux monitor is calibrated by relating the total rate of neutrons striking the Alpha-Gamma device to the event rate seen in the neutron flux monitor when it is placed upstream of the Alpha-Gamma device on the same beamline. Expressed according to deposit usage, the efficiency of the neutron flux monitor in terms of measured quantities is given by

(2) $$\epsilon_{0}=\frac{r_{\alpha ,t}}{r_{\gamma }(\text{thick})}\frac{r_{\gamma }(\text{thin})}{r_{\alpha }(\text{thin})}\frac{r_{\alpha }(\text{Pu})}{R_{\alpha }(\text{Pu})}\frac{\lambda_{o}}{\lambda_{\text{mono}}},$$

where *r_α,t_* is the rate of alphas plus tritons detected in the flux monitor on a monochromatic beam whose mean wavelength is *λ*_mono_, *λ_o_* = 0.1798 nm is defined to be the wavelength of a 2200 m/s neutron, *r_γ_* (thick) is the rate of gammas detected from the neutron capture reaction on ^10^B at the same time as *r_α,t_*, *r_γ_* (thin) is the observed gamma rate in the thin-deposit configuration, *r_α_*(thin) is the alpha rate observed at the same time, *R_α_*(Pu) is the precisely known alpha activity of a ^239^Pu source, and *r_α_*(Pu) is the alpha rate observed when the Pu source is inserted into the device. An overall relative uncertainty of 0.06% has been achieved [10, 11].

The Alpha-Gamma device was used to update the result of the 2005 neutron lifetime measurement [27] in 2013 [11], reducing the neutron counting efficiency uncertainty from 2.7 s to 0.5 s. In the new run of the lifetime experiment, several additional improvements related to neutron counting are being done. The Alpha-Gamma device will be operated simultaneously with the neutron lifetime experiment, allowing us to calibrate the neutron flux monitor before and after lifetime data acquisition. This will check that there are no changes to the efficiency during the course of the measurement. Additionally, we plan to run with multiple deposits in the neutron monitor. BL1 used one ^6^Li deposit in the neutron monitor, a 40 μg/cm^2^ deposit. For BL2, we plan to use 40 μg/cm^2^, 30 μg/cm^2^, and 20 μg/cm^2^6Li deposits. In addition to serving as a further check on systematics, this will also allow us to reduce the correction and uncertainty due to the absorption of neutrons by the ^6^Li deposit. This was one of the largest corrections applied in the 2005 result, at 5.2 s with a 0.8 s uncertainty. This correction roughly scales with the deposit mass so a 20 μg/cm^2^ deposit will have a correction and associated uncertainty that is roughly half of what it was for the 40 μg/cm^2^ deposit used in the previous measurement. In addition to running with multiple ^6^Li deposits, we also have the option to use various ^10^B deposits in the neutron monitor. We also anticipate taking data with the deposit facing both upstream and downstream as a further check on various corrections. Note that neutron scattering in the ^6^Li deposit is negligible compared with the absorption while scattering from the Si backing can be more significant and will be measured separately to determine the necessary correction. Finally, we have measured the NG-C neutron wavelength distribution at a position just upstream of the magnet bore (after our final beam collimation aperture). This measurement was compared to and agrees quite well with simulations. A more precise knowledge of the neutron wavelength distribution serves to also improve the correction for absorption of the neutrons by the ^6^Li deposit.

## 4. Proton counting

Since the lifetime apparatus was last operated nearly two decades ago, we have a better understanding of most of the large remaining systematic effects and proton counting uncertainty. For example, simulations showed that the magnetic field gradient was too large for the longest trap length that was used in the 2001 data set [27]. It necessitated a 5.3 s correction with a 0.8 s uncertainty. In the current experiment, this correction is reduced to about 1 s, with a ≈0.2 s uncertainty, by decreasing the maximum trap length used by 10%. Additionally, the superconducting solenoid and proton detector system were used in two subsequent experiments to measure radiative neutron decay [30–32], for which they were modeled extensively using GEANT4 [33, 34] and MCNP packages as well as individually-developed codes [35]. This has given us a much more sophisticated understanding of the magnetic and electrostatic effects, geometry, and particle transport.

The radiative decay experiments also demonstrated that one could operate a proton detector at high voltage with larger area (600 mm^2^ versus 300 mm^2^) and thicker (1.5 mm versus 0.3 mm) silicon detectors. The larger area enables us to study and reduce the uncertainty from protons potentially lost due to neutron halo, that is, decay protons that are transported to the detector but strike outside the active area of the silicon. Such tests are currently underway. The thicker depletion region reduces the detector capacitance and thus the leakage current (noise) of the detector. The increased noise due to increased sensitivity to background radiation is negligible compared to the reduction in electronic noise. One can thus lower the energy threshold for proton detection and reduce the uncertainty in the extrapolation for backscattered protons.

Additional simulations are being performed explicitly for the BL2 experiment. A neutron ray-tracing program for the BL2 collimation using realistic neutron phase space of the NG-C beamline and including the effects of gravity provides wavelength-dependent neutron profiles at the ^6^Li deposit as well as proton birth positions within the trap. These simulations have been benchmarked against wavelength and spatial-profile measurements of the neutron beam. A GEANT4 simulation of the previous radiative-decay experiment, which includes the magnetic and electric fields, has been modified to take as input these proton starting positions. The effect of proton backscattering on proton detection efficiency has been studied for different detector configurations, varying dead-layer material (Au, Si) and thickness, as well as detector high-voltage bias. The advantage of the GEANT4 simulations over the previous SRIM [36] calculations of proton backscattering is that GEANT4 can track the proton after backscattering, and thus can determine the fraction that do not return to the active region for each detector configuration. The backscatter fraction was measured by extrapolation in BL1. A series of electric fields for different conditions of the proton trap are currently being modeled in COMSOL® [37]. These will be used in the GEANT4 simulation to study effects of the spatial profile of proton birth locations on the proton detection rate as a function of different field settings of the trap as the length of the trap is varied in the lifetime experiment. It is understood that it is very difficult to quantify uncertainties from the simulation codes for low energy neutron decay products. As such, our approach is to perform as many cross checks as possible and also to benchmark simulation results wherever possible.

We have also improved several aspects of the electronics since 2001. In particular, a lower-noise preamplifier is operating successfully. It enables a lower energy threshold for proton detection, leading to better understanding of the low-energy proton tail. It also allows for operation at lower proton acceleration voltages thereby extending the range of voltages available. These features will reduce the uncertainty associated with the proton detection efficiency. A new data acquisition system can digitize all proton waveforms from both the preamplifier and the shaping amplifier, thus making it possible to study multiple-proton and background events in detail. Furthermore, new data analysis methods have been developed that utilize the digitized waveforms. The analysis methods will be discussed in more detail in Sect. 5.

**Figure 2.**
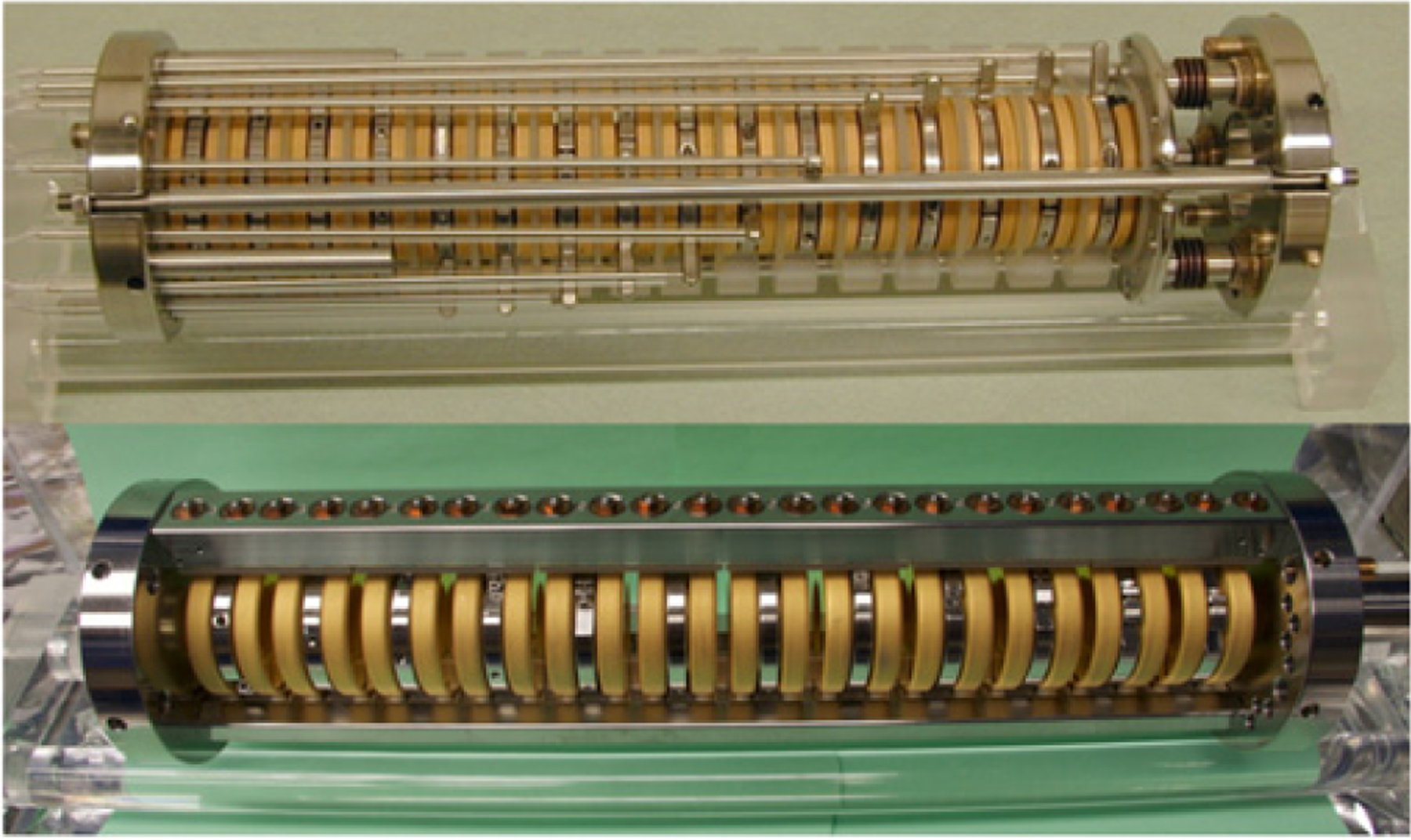
Photographs of electrode stacks for the mark II (top) and mark III (bottom) traps.

A new version of the proton trap, dubbed mark III, has been developed and tested. Photos of the two trap electrode stacks are shown in Fig. 2. The old trap (“mark II”) consists of a stack of gold-plated fused quartz trap electrodes, with fused quartz spacers between each electrode. The whole stack is held together by axial compression from two end plates. The mark III trap eliminates the need for the spacers by holding each electrode individually in a kinematic mount, with the whole structure held together by a stainless steel frame. The advantages of the mark III trap include better pumping of the trap volume, better surface finish on the interior electrode surface, and easier metrology of the fully assembled trap. Off-line testing has demonstrated satisfactory performance of the mark III trap. Using both versions of the trap to take data will give us yet another handle on systematic effects. Additionally, the new trap allows for a data blinding scheme utilizing the trap electrode lengths as the blind.

Extensive off-line testing of the proton trapping and detection system has been performed to study the instabilities seen in earlier versions of the experiment and achieve stable operation. These instabilities plagued the earlier run of the experiment and seriously hampered efforts to study systematic effects. We have been able to consistently run in a more stable configuration and have access to a significantly wider range of parameter space than was accessible previously. Three important issues related to system stability have been identified. One, the quality of the electrical connection to the detector is critical. This connection is made by an SMA to microdot adapter. Ensuring that this connection is clean and tight is essential to trouble-free operation. Two, the quality of the detector surface matters, and it degrades with each successive spark. Three, having a good vacuum, including low helium partial pressure, inside the trap and detector region is critical. These last two points will be discussed in more detail below.

The surface of a silicon detector comes out of the package fairly pristine, although it begins to accumulate some small amount of dust simply from exposure to a non-clean-room environment. Although cause and effect are not entirely understood, we have seen that detectors that have had major sparks come out of the vacuum chamber significantly dustier than detectors that have been operating smoothly. Further, detailed microscope inspections have identified various types of surface damage that can arise and accumulate with sparking events. As some of this surface damage could potentially cause “blind” spots or other issues with the detectors, detectors that have had one or more sparks that caused the high voltage to trip are removed from further service even if they otherwise behave satisfactorily. While detector sparks are generally random under normal operating conditions, we have seen that they are more likely to occur the longer a detector is in continuous operation. In order to minimize sparks and maintain quality detector surfaces, we have set a limit of 3 weeks (half of a standard reactor cycle) of continuous operation for a detector before it is removed and swapped for a new detector or cleaned with a puff of nitrogen gas and put back into operation with cleaned and re-made connections.

High vacuum inside the trap and proton detector region is critical. Repeated tests have shown that even relatively modest increases in residual gas pressure can lead to an excess of proton-like signals and/or detector sparking and failure. Additionally, these tests suggest that an increase in the partial pressure of helium may be particularly problematic. As the proton trap and detector are located inside the cryogenic magnet bore, it is difficult to determine the exact pressure at the trap location. A pressure gauge and a residual gas analyzer are located just upstream of the magnet bore, between the magnet bore and an ion pump. Since the cryogenic magnet bore also serves as a large and very effective cryo-pump, we assume that the pressure near the trap is significantly lower than the readings on the external pressure gauge. To maintain high vacuum, we bake all bakeable portions of the vacuum system prior to the magnet cool-down, repeating the bake of the portions that get vented during a detector swap after each detector swap. This allows us to keep the pressure at our gauge below 1 × 10^−6^ Pa, and typically below 5 × 10^−7^ Pa (the lower limit of our gauge). In this manner we see consistently stable and low-noise operation of the proton trap and detection system.

One additional improvement of BL2 over BL1 is the move of the apparatus from the NG-6 beamline to the NG-C beamline at the NIST Center for Neutron Research (NCNR). While the NG-C beamline has a higher cold neutron flux than NG-6, the collimation necessary to keep the neutrons (and thus decay protons) within the area of our detectors prevents us from utilizing much of the increase in flux. However, some improvement comes from the fact that NG-C is a curved guide with no direct line-of-sight back to the reactor core. This greatly reduces the gamma background and eliminates the need to add the Bi filter in the beam that was required for BL1. The statistical uncertainty on the previous lifetime result was 1.2 s. Because it was clear that the final result would be dominated by the neutron counting systematic, there was no point in spending beam time to reduce the statistical uncertainty. In the current experiment, significantly more beam time will be devoted to improving the statistics. We expect that the apparatus will achieve a statistical uncertainty in proton counting at or below 0.5 s.

## 5. Analysis

**Figure 3.**
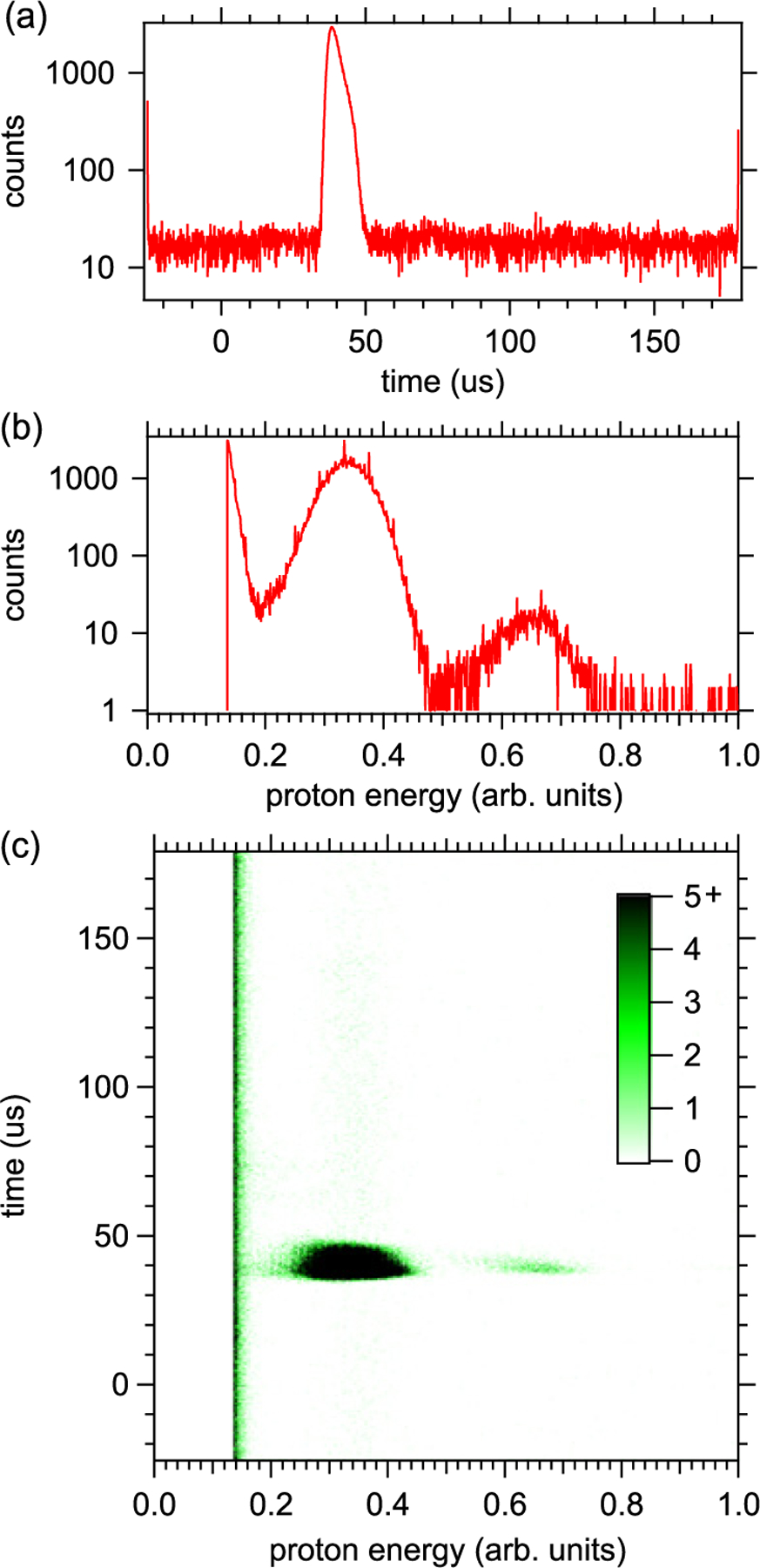
Example 1-dimensional timing (a) and energy (b) histograms and a 2-dimensional histogram showing the counts versus timing and energy (c).

The ability to digitize all proton waveforms, both the preamplifier signal and the shaped amplifier signal, has opened up new data analysis possibilities that were not available in BL1. We are currently using two completely independent data analysis methods. The first method, which is similar to the method used in BL1, makes use of the shaped signals. This method takes each waveform and records the time and peak height (energy) of the highest point in the waveform. These data are used to make 1-D and 2-D histograms of the proton arrival time and proton energy. An example of these histograms can be seen in Fig. 3. Typically, the 1-D timing spectrum is used to count the number of protons detected, with a correction using Poisson statistics made for the probability of detecting more than one proton. Other types of cuts and analyses are used to study certain systematic effects.

**Figure 4.**
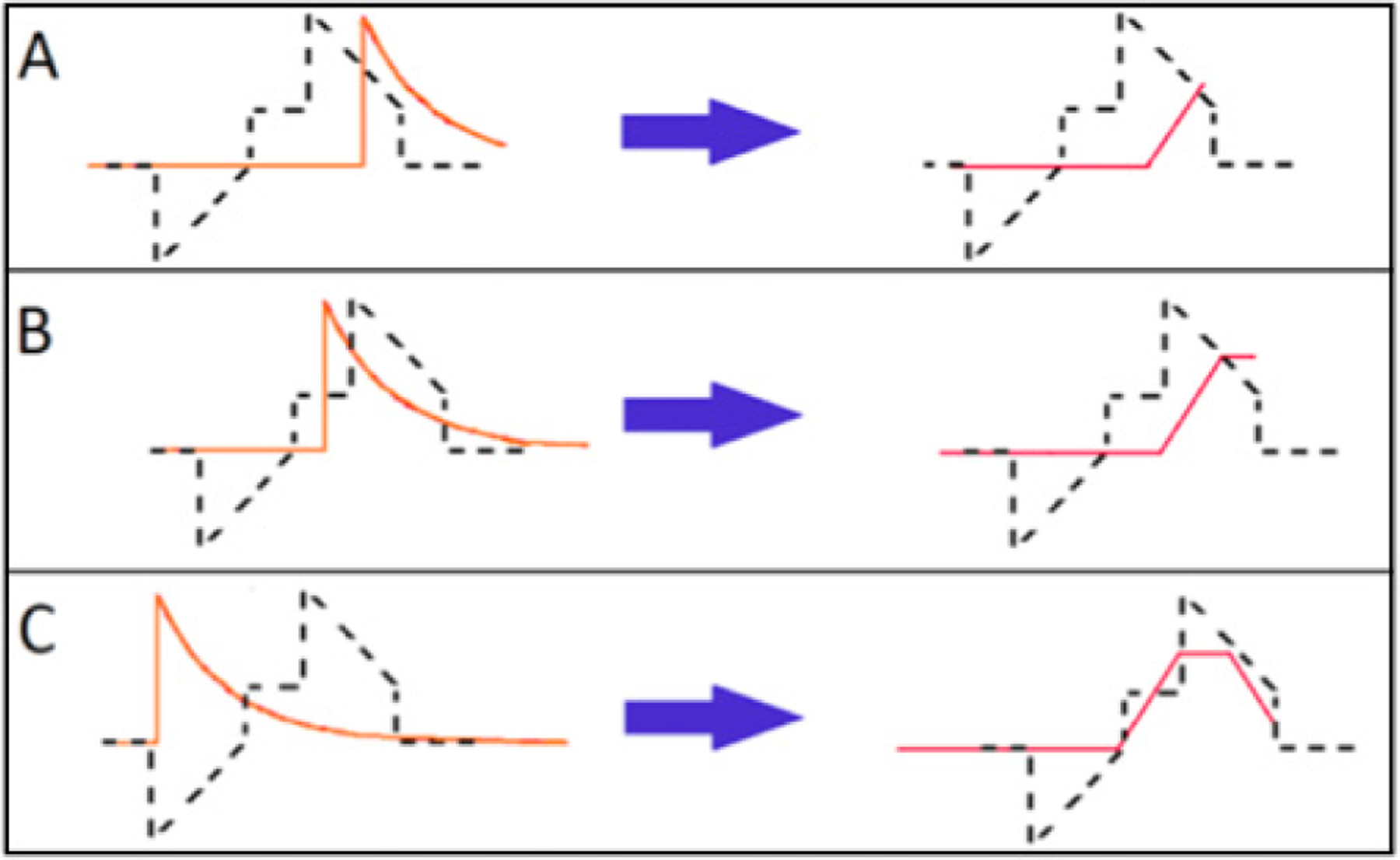
Example of trapezoid filter shaping response function (dashed line). The trapezoid filter input (preamplifier signal) is shown on the left (solid orange line), the output of the filter is shown on the right (solid red line). A, B, and C show the response function moving across the input function.

**Figure 5.**
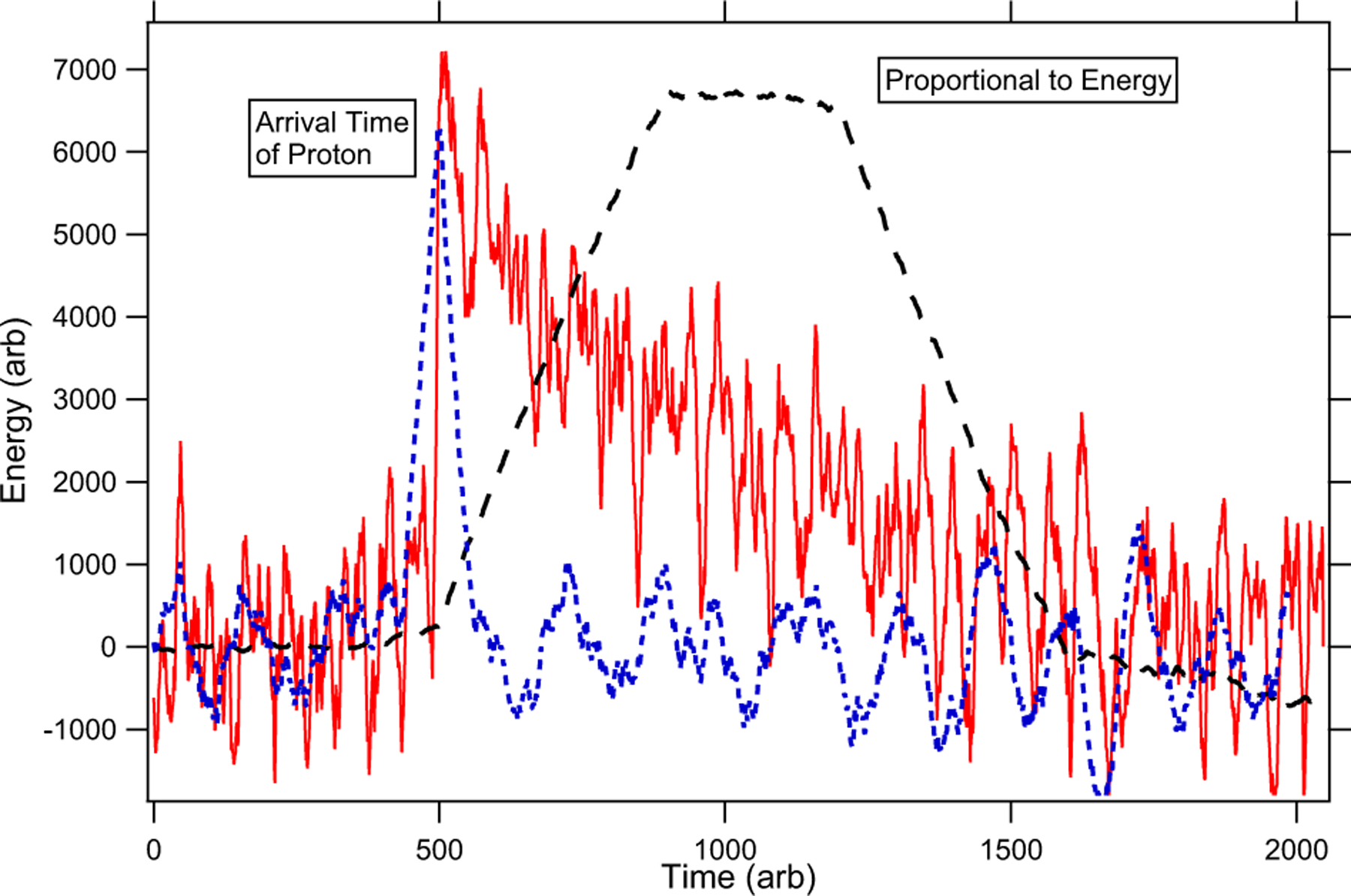
An example of the preamplifier proton pulse (solid red) and the output of the trapezoid filter optimized for energy extraction (dashed black) or time resolution (dotted blue).

The second, and new, analysis method utilizes a finite impulse response trapezoid filter on the digitized preamplifier waveforms. This method convolves a shaping response function [38] with the preamplifier signal to create an output waveform in the shape of a trapezoid. An example of the shaping response function and resulting trapezoid can be seen in Fig. 4. Energy and timing information is retained from the original preamplifier waveform, and the filter can be optimized for either energy resolution or timing resolution as shown in Fig. 5. This filter allows for pulse shape discrimination to be done on a waveform-by-waveform basis. This is done by extracting the height of the trapezoid (energy) as well as the positive and negative slopes of the sides of the trapezoid. This analysis allows for the direct identification of multiple proton events, and the study of systematic effects.

These independent methods offer differing advantages and issues; the consistency between them is a useful tool in ensuring that the data are well understood and that all protons are accounted for.

## 6. Progress and outlook

The BL2 lifetime experiment is currently running on the NG-C beamline at the NCNR. The effort to establish reliable absolute neutron counting at a level below 1 s is complete, although we continue to investigate systematic effects associated with neutron counting. The independent calibration of the neutron flux monitor efficiency removes the largest systematic uncertainties that have precluded a more precise lifetime measurement using a cold neutron beam; specifically it eliminates the reliance on a cross section evaluation. Subsequent experience with the neutron lifetime apparatus has provided valuable insights into ways to reduce the proton counting uncertainty as well.

A series of early checks and tests has been completed and we are now running in production mode utilizing the mark II trap. The main focus of this experiment is identifying and testing as many systematic effects as possible. The improved stability of operation and wider range of running parameters enables us to cover a much wider range of systematic checks than was previously possible. Specifically, we have demonstrated the ability to run with trapping times ranging from 3 ms to 30 ms (while BL1 was largely limited to 10 ms, with only one run taken at 5 ms), and we are investigating the possibility of extending the trapping time beyond 30 ms. We have taken data at the full range of trap lengths. We have also taken data at a range of acceleration potentials up to 30 kV, again with the possibility of going higher. Given the improvements in neutron counting and proton counting outlined above, along with the wide range of parameter space available in which we can run, we anticipate making a measurement at a precision of 1 s or better, with a significantly larger set of checked systematic effects than were addressed in the previous results.
